# Current Role of SLGT2 Inhibitors in the Management of the Whole Spectrum of Heart Failure: Focus on Dapagliflozin

**DOI:** 10.3390/jcm12216798

**Published:** 2023-10-27

**Authors:** Carlos Escobar, Domingo Pascual-Figal, Luis Manzano, Julio Nuñez, Miguel Camafort

**Affiliations:** 1 Cardiology Department, University Hospital La Paz, 28046 Madrid, Spain; 2 Cardiology Department, Hospital Clinico Universitario Virgen de la Arrixaca, 30120 Murcia, Spain; dpascual@um.es; 3 Spanish National Cardiovascular Research Centre (CNIC), 28029 Madrid, Spain; 4 Department of Medicine, Universidad de Murcia, 30100 Murcia, Spain; 5 Internal Medicine Department, University Hospital Ramon y Cajal, Alcala de Henares University, 28034 Madrid, Spain; luis.manzano@uah.es; 6 Cardiology Department, University Hospital Clínico of Valencia, Instituto de Investigación Sanitaria (INCLIVA), 46010 Valencia, Spain; yulnunez@gmail.com; 7 CIBER Cardiovascular, 28029 Madrid, Spain; 8 Internal Medicine Department, Hospital Clinic, University of Barcelona, 08036 Barcelona, Spain; camafort@clinic.cat; 9 CIBER OBN, ISCIII (Centro de Investigación Biomédica en Red, Instituto de Salud Carlos III), 28222 Madrid, Spain; 10 Working Group of Cardiovascular Risk, Nutrition, and Aging, IDIBAPS (Instituto de Investigaciones Biomédicas August Pi i Sunyer), 08036 Barcelona, Spain

**Keywords:** dapagliflozin, heart failure, hospitalization, mortality

## Abstract

Heart failure (HF) is associated with a high morbidity and mortality burden. In light of more recent evidence, SGLT2 inhibitors are currently recommended as first-line therapy in managing patients with HF, regardless of ejection fraction, to reduce HF burden. The DAPA-HF and DELIVER trials, and particularly, the pooled analysis of both studies, have shown that dapagliflozin significantly reduces the risk of cardiovascular death, all-cause death, total HF hospitalizations, and MACE in the whole spectrum of HF, with sustained benefits over time. Recent data have shown that the full implementation of dapagliflozin in clinical practice would translate into a robust reduction in hospitalizations for HF and death in real-life populations. Many pathophysiological mechanisms have been involved in these benefits, particularly the positive effects of dapagliflozin on reversing cardiac (atrial and ventricular) remodeling, reducing cardiac fibrosis and inflammation, and improving endothelial dysfunction. In this manuscript, we reviewed from a practical point of view the role of dapagliflozin in the management of the whole spectrum of patients with HF.

## 1. Introduction

Heart failure (HF) is a very common syndrome worldwide. In the general adult population, the current prevalence of HF is around 2% but increases with age, reaching 7% in those patients >45 years and 16% in those over 75 years [1,2,3]. Of note, due to the aging of the population and the better management of acute cardiovascular conditions, it is very likely that these numbers will increase in the following years [4].

Despite traditional treatments with renin-angiotensin system inhibitors and beta blockers, death rates of patients with HF have remained unacceptably high (i.e., 40% after a median follow-up of 2.5 years) [5]. Fortunately, these numbers are improving, particularly in those countries with a higher implementation of guideline-directed medical therapy [6,7]. Along this same line, a meta-analysis of three studies, EMPHASIS-HF trial (Eplerenone in Mild Patients Hospitalization and Survival *Study* in Heart Failure), PARADIGM-HF trial (Prospective Comparison of ARNI [Angiotensin Receptor–Neprilysin Inhibitor] with ACEI [Angiotensin-Converting–Enzyme Inhibitor] to Determine Impact on Global Mortality and Morbidity in Heart Failure Trial) and DAPA-HF trial (Dapagliflozin and Prevention of Adverse Outcomes in *Heart Failure*) showed that compared with the combination of an angiotensin-converting enzyme inhibitor (ACEi) or an angiotensin receptor blockers (ARB) and a beta blocker, the combination of an angiotensin receptor-neprilysin inhibitor (ARNI), a beta blocker, eplerenone (a mineralocorticoid receptor antagonist) and dapagliflozin (a sodium-glucose cotransporter 2 inhibitor [SGLT2i]) was associated with a significant 50% risk reduction of cardiovascular death and a significant 47% risk reduction of all-cause mortality [8].

Acute HF represents the first cause of hospitalization in patients over 65 years in developed countries [3]. HF hospitalizations are an inflection point in the evolution and progression of the disease. Thus, in-hospital mortality varies between 4% and 10% and mortality within the first year after discharge reaches 25–30% of cases [9,10]. In addition, the risk of rehospitalization is high, particularly during the vulnerable period that accounts for the first months after discharge [11]. The meta-analysis of the EMPHASIS-HF, PARADIGM-HF and DAPA-HF trials showed that compared with the combination of an ACEi/ARB and a beta blocker, the quadruple therapy with ARNI, beta blocker, a mineralocorticoid receptor antagonist and dapagliflozin significantly reduced the risk of cardiovascular death or HF hospital admission by 62% and also hospital admission for HF alone by 68% [8]. Furthermore, HF has an important impact on the health system’s economic burden, making HF hospitalizations the main determinant for total costs. As a result, reducing HF hospitalization may translate into a reduction in health care costs [12,13].

There are different phenotypes of HF according to ejection fraction. Thus, around 40–50% of patients may have HF with reduced ejection fraction (HFrEF), 40% HF with preserved ejection fraction (HFpEF), and around 10% HF with mildly reduced ejection fraction (HFmrEF) [1,14,15]. Despite the substantial differences in the clinical profile, comorbidities, and cause of death according to HF phenotypes, the risk of adverse clinical outcomes, both cardiovascular death and HF (re-)hospitalization are quite similar along the whole spectrum of left ventricular ejection fraction [1,14,15].

The pathogenesis of HF is complex, as different neurohormonal systems are involved, including the overactivation of deleterious pathways, such as the renin-angiotensin and the sympathetic nervous systems, but also the inhibition of protective pathways, such as the natriuretic peptides system and the nitric oxide-soluble guanylate cyclase-cGMP system [16,17,18,19]. SGLT2i promotes glucosuria, osmotic diuresis and natriuresis, improves volume status and cardiac metabolism, sodium-hydrogen exchange in the myocardium, reduces cardiac fibrosis, prevents and reverses adverse cardiac remodeling through the reduction of apoptosis, necrosis and autophagy and the improvement of myocardial oxygen supply/demand, reduce sympathetic stimulation, modulate the levels of leptin and adiponectin, improve control of risk factors, preserve renal function and decrease inflammation and oxidative stress [20,21,22,23,24,25,26,27,28].

**Figure 1:**
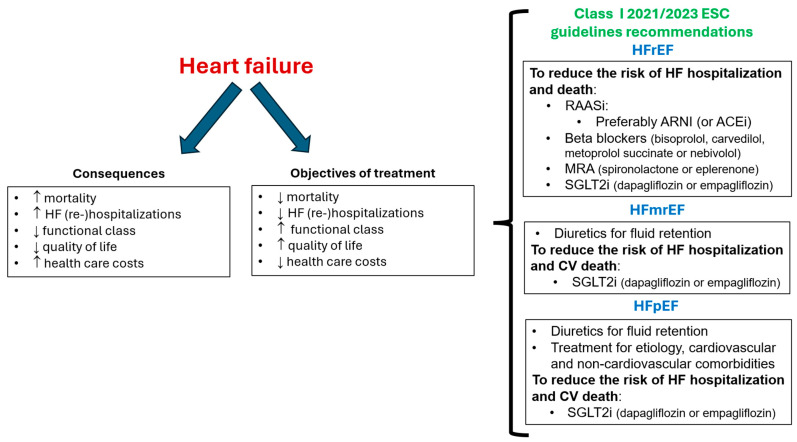
Objectives of treatment of heart failure and class I ESC guidelines recommendations. ARNI: angiotensin receptor-neprilysin inhibitor; CV: cardiovascular; ESC: European Society of Cardiology; HFmrEF: HF with mildly reduced ejection fraction; HFpEF: HF with preserved ejection fraction; HFrEF: HF with reduced ejection fraction; MRA: mineralocorticoid receptor antagonists; SGLT2i: sodium-glucose cotransporter 2 inhibitor; RAASi: renin-angiotensin system inhibitor. Figure performed with data from references [5,6,7,8,9,10,11,12,29,30].

The objectives of HF management should include reducing the risk of cardiovascular death and HF hospitalization and improving symptoms and quality of life [29,30]. In this context, current guidelines recommend as first-line therapy the quadruple therapy with renin-angiotensin system inhibitors, preferably with ARNI, beta-blockers, mineralocorticoid receptor antagonists (either spironolactone or eplerenone), and SGLT2i for patients with HFrEF. More recently, the use of diuretics for fluid retention and SGLT2i (dapagliflozin/empagliflozin) is also indicated for patients with HFmrEF and HFpEF [29,30,31] (Figure 1).

Unfortunately, adherence to evidence from practitioners and to mediation from patients remains a problem in clinical practice, which probably translates into a higher risk of events [1,15,32]. Otherwise, it seems that SGLT2i may facilitate the tolerance and persistence for other evidence-based mediations [32].

In this review, we practically update the role of dapagliflozin in the management of the whole spectrum of patients with HF.

## 2. Dapagliflozin: Beyond HF Protection

Dapagliflozin is rapidly absorbed after oral administration (Cmax of 2 h), has an absolute oral bioavailability of 78%, and can be taken with or without food. Dapagliflozin is approximately 91% protein bound. Of note, the CYP-mediated metabolism of dapagliflozin is minor, which leads to a low risk of drug-drug interactions. The mean plasma terminal half-life is around 13 h and is mainly eliminated through urinary excretion (75% in urine and 21% in feces). The dose of dapagliflozin (10 mg once daily) is not required to be adjusted according to renal or hepatic function, age, gender, race, or body weight [33,34,35].

The benefits of dapagliflozin have been proven not only in patients with HF but also in patients with type 2 diabetes and chronic kidney disease, providing a wide spectrum of cardiovascular protection. The DECLARE–TIMI 58 (Dapagliflozin Effect on Cardiovascular Events–Thrombolysis in Myocardial Infarction 58) trial included 17,160 patients with type 2 diabetes who had (40.6%) or were at risk (59.4%) for atherosclerotic cardiovascular disease. After a median follow-up of 4.2 years, compared to placebo, treatment with dapagliflozin was non-inferior with respect to MACE, a composite of cardiovascular death, myocardial infarction, or ischemic stroke (hazard ratio [HR] 0.93; 95% confidence interval [CI] 0.84–1.03), but was superior when considering those patients with prior myocardial infarction (HR 0.84; 95% CI 0.72–0.99) [36,37]. Remarkably, dapagliflozin significantly reduced the risk of cardiovascular death or hospitalization for HF (HR 0.83; 95% CI 0.73–0.95), largely due to a reduction in HF hospitalizations (HR 0.73; 95% CI 0.61–0.88) and the renal composite of ≥40% decrease in estimated glomerular filtration rate (eGFR) to <60 mL/min/1.73 m^2^, new end-stage renal disease, or death from renal or cardiovascular causes (HR 0.76; 95% CI 0.67–0.87) [36].

The DAPA-CKD (Dapagliflozin and Prevention of Adverse Outcomes in Chronic Kidney Disease) trial included 4304 individuals with an eGFR of 25 to 75 mL/min/1.73 m^2^ and a urinary albumin-to-creatinine ratio of 200 to 5000 mg/g. After a median follow-up of 2.4 years (the trial was prematurely stopped because of the overwhelming efficacy of dapagliflozin), compared to placebo (on top of renin-angiotensin system inhibition), dapagliflozin significantly reduced the risk of the primary outcome composed of a sustained decline in the eGFR ≥ 50%, end-stage kidney disease, or death from renal or cardiovascular causes by 39% (HR 0.61; 95% CI 0.51–0.72), the risk of death from cardiovascular causes or HF hospitalization by 29% (HR 0.71; 95% CI 0.55–0.92) and all-cause death by 31% (HR 0.69; 95% CI 0.53–0.88) [38]. Dapagliflozin also decreased the risk of major adverse kidney and cardiovascular events and mortality for any cause regardless of the presence of type 2 diabetes [39]. The benefits of dapagliflozin were maintained even in those patients with stage 4 chronic kidney disease [40]. Moreover, dapagliflozin reduced the risk of kidney failure and cardiovascular death or HF hospitalization and improved survival in patients with chronic kidney disease, regardless of the baseline presence of prior HF [41].

## 3. Dapagliflozin and HF

Although the most important clinical trials that have assessed the role of dapagliflozin among the HF population are the DAPA-HF and DELIVER (Dapagliflozin Evaluation to Improve the Lives of Patients with Preserved Ejection Fraction Heart Failure) trials [42,43], many other studies have been involved in the HF clinical development of dapagliflozin. For example, the DEFINE-HF (Dapagliflozin Effects on Biomarkers, Symptoms, and Functional Status in Patients with HF with Reduced Ejection Fraction) trial was an investigator-initiated, multicentre, randomized controlled trial of 263 patients with NYHA II-III HFrEF, eGFR ≥ 30 mL/min/1.73 m^2^, and elevated natriuretic peptides. Patients received dapagliflozin 10 mg daily or placebo for 12 weeks. Although natriuretic peptide levels were not significantly modified by dapagliflozin, there were significantly more patients experiencing clinically meaningful improvements in HF-related health status assessed by the Kansas City Cardiomyopathy Questionnaire or natriuretic peptides, regardless of type 2 diabetes status [44]. In addition, this study suggested a direct effect of dapagliflozin on more effective “decongestion”, as dapagliflozin improved lung fluid volumes measured by remote dielectric sensing after 12 weeks of therapy [45]. A pooled patient-level analysis from DEFINE-HF (263 patients with symptomatic HFrEF) and PRESERVED-HF (Effects of Dapagliflozin on Biomarkers, Symptoms and Functional Status in Patients with Preserved Ejection Fraction Heart Failure that included 324 patients with symptomatic HFpEF and elevated natriuretic peptides) trials showed that after 12 weeks of treatment, dapagliflozin improved symptoms and reduced physical limitations across the full range of ejection fraction [46]. The evidence, supporting the role of dapagliflozin on short-term maximal functional capacity was assessed in the DAPA-VO2 randomized clinical trial. This study showed that treatment with dapagliflozin led to a significant short-term improvement in peak oxygen consumption at 1 and 3 months in patients with stable HFrEF, compared to placebo [47].

### 3.1. Dapagliflozin and HFrEF: The DAPA-HF Trial

DAPA-HF was a phase 3, placebo-controlled trial that included 4744 patients with symptomatic HFrEF (left ventricular ejection fraction ≤ 40%) and elevated natriuretic peptides. Patients with type 1 diabetes, a systolic blood pressure <95 mmHg, and an eGFR < 30 mL/min/1.73 m^2^ were excluded from the study. At baseline, the mean age was 66 years, 24% were women, two-thirds were in NYHA functional class II, 42% had type 2 diabetes, and 41% had an eGFR < 60 mL/min/1.73 m^2^. Regarding baseline HF treatments, around 95% were taking renin-angiotensin system inhibitors (11% ARNI), 96% beta blockers, and 71% mineralocorticoid receptor antagonists [42].

**Figure 2:**
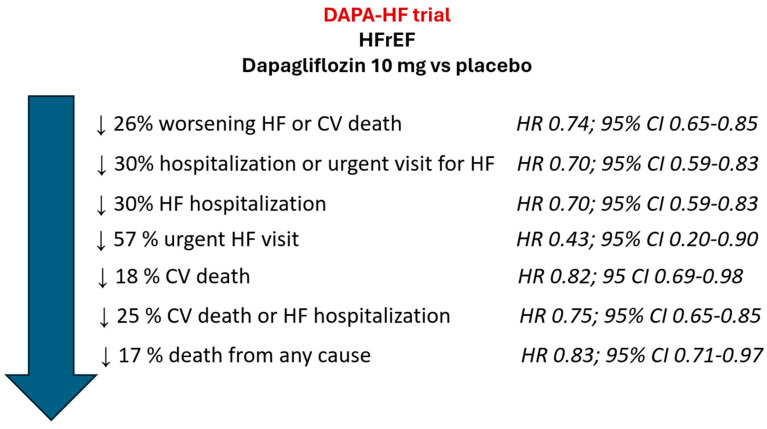
Main results of DAPA-HF trial. CI: confidence interval; CV: cardiovascular; HF: heart failure; HFrEF: HF with reduced ejection fraction; HR: Hazard Ratio. Figure performed with data from reference [42].

After a median follow-up of 18.2 months, compared to placebo, dapagliflozin significantly reduced the risk of the primary combined endpoint of worsening HF (hospitalization or an urgent visit resulting in intravenous therapy for HF) or cardiovascular death by 26% (HR 0.74; 95% CI 0.65–0.85). Similarly, dapagliflozin significantly reduced the risk of a first worsening HF event, and death from any cause (Figure 2). These results were independent of type 2 diabetes status.

From baseline to 8 months, HbA1c decreased −0.21% (vs. +0.04% in the placebo group; treatment difference −0.24%; *p* < 0.001), systolic blood pressure −1.92 mmHg (vs. −0.38 mmHg in the placebo group; treatment difference −1.27 mmHg; *p* = 0.002), and body weight −0.88 Kg (vs. +0.10 Kg in the placebo group; treatment difference −0.87 Kg; *p* < 0.001). Remarkably, the incidence rates of volume depletion (7.5%), renal adverse event (6.5%), and major hypoglycemia (0.2%) were low with dapagliflozin and similar to the placebo group [42].

**Figure 3:**
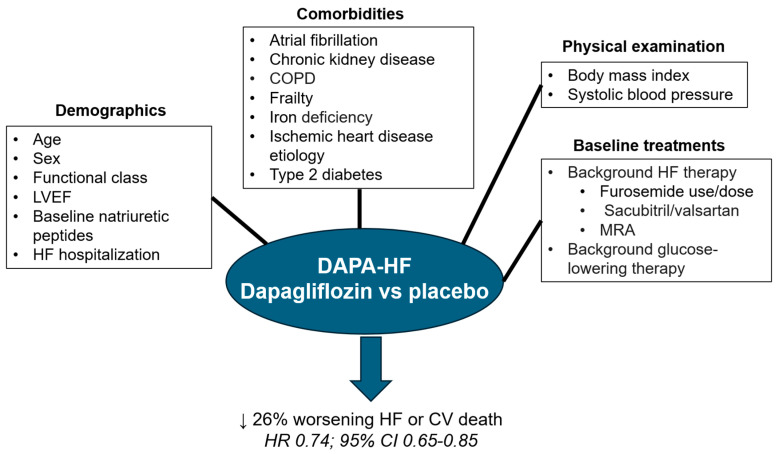
The relative benefits of dapagliflozin vs. placebo on the primary outcome in the DAPA-HF trial were independent of demographics, comorbidities, physical examination and background therapies. CI: confidence interval; COPD: chronic obstructive pulmonary disease; CV: cardiovascular; HF: heart failure; LVEF: left ventricular ejection fraction; MRA: mineralocorticoid receptor antagonist; HR: Hazard Ratio. Figure performed with data from references [42,48,51,52,53,54,55,56,57,58,60,61,62,63,64,65,66,68].

Many prespecified sub-studies and post hoc analyses of the DAPA-HF trial have been published and have provided relevant data that complete the information provided by the DAPA-HF trial (Figure 3).

Although patients with type 2 diabetes had a higher risk of events in the DAPA-HF trial, the relative benefit of dapagliflozin vs. placebo was independent of diabetes status (primary outcome: without diabetes HR 0.73; 95% CI 0.60–0.88; with diabetes HR 0.75; 95% CI 0.63–0.90: *p*-value for interaction = 0.80) [48]. In addition, an exploratory analysis from the DAPA-HF trial showed a 32% risk reduction in the incidence of diabetes (HR 0.68; 95% CI 0.50–0.94). This was more evident in those patients with prediabetes at baseline (HbA1c 5.7–6.4%) [49]. Furthermore, in a pooled analysis of the DAPA-CKD and DAPA-HF trials, treatment with dapagliflozin was associated with a significant reduction of 33% in the risk of new-onset diabetes after a median follow-up of 21.2 months (HR 0.67; 95% CI 0.51–0.88) [50]. Remarkably, those patients who developed diabetes in the DAPA-HF trial had a higher risk of adverse events [49].

Ischemic heart disease represents the most important cause of HFrEF [29]. In the DAPA-HF trial, 56.4% of patients had an ischaemic etiology. Compared with placebo, dapagliflozin reduced the risk of the primary outcome to a similar extent, regardless of ischemic etiology (ischemic: HR 0.77; 95% CI 0.65–0.92, non-ischemic HR 0.71; 95% CI 0.58–0.87, P for interaction = 0.55). Improvement of symptoms similarly occurred with dapagliflozin independently of the etiology, as well as safety [51]. Moreover, the benefits of dapagliflozin were consistent across the broad spectrum of baseline risk according to the MAGGIC risk score [52].

The presence of renal disease is common in patients with HFrEF and makes more difficult the management of these patients, particularly with the use of some drugs, such as renin angiotensin aldosterone system inhibitors [29]. A specific analysis of DAPA-HF data according to baseline kidney function (41% of patients had an eGFR < 60 mL/min/1.73 m^2^ at baseline) showed that the relative benefits of dapagliflozin did not differ according to baseline renal function (i.e., primary endpoint (eGFR < 60 vs. ≥60 mL/min/1.73 m^2^: HR 0.71; 95% CI 0.59–0.86 vs. HR 0.77; 85% CI 0.64–0.93, respectively; P for interaction = 0.54). In addition, the decline in renal function over the follow-up was lower with dapagliflozin. These results were independent of diabetes status [53].

Another important question is whether the effects of dapagliflozin vary according to age, particularly in the elderly. In the DAPA-HF trial, although the rates of the primary endpoint increased with age (<55 years: 13.6; 55 to 64 years: 15.7; 65 to 74 years: 15.1; ≥75 years: 18.0 per 100 person-years in the placebo arm), the relative benefit of dapagliflozin remained across all age spectrum (HR 0.87; 95% CI 0.60–1.28, HR 0.71; 95% CI 0.55–0.93, HR 0.76; 95% CI 0.61–0.95, and HR 0.68; 95% CI, 0.53–0.88, respectively; P for interaction = 0.76). These numbers were also consistent regarding other efficacy endpoints, as well as the safety profile [54]. In this context, it is also important to determine the efficacy and safety of dapagliflozin in frail patients. In the DAPA-HF trial, 50.4% of patients were not frail, 33.9% were frailer, and 15.7% were most frail, according to the frailty index. Although dapagliflozin improved all efficacy and safety outcomes, regardless of frailty status, the absolute reductions were greater in more frail patients, indicating that dapagliflozin can be safely prescribed even in more frail patients [55].

Dapagliflozin may slightly reduce systolic blood pressure levels [34]. Of note, in the DAPA-HF trial, patients with a systolic blood pressure <95 mmHg were excluded from the study [42]. From baseline to 2 weeks, dapagliflozin only reduced systolic blood pressure by 2.54 mmHg. No more discontinuation of treatment rates was observed with dapagliflozin according to systolic blood pressure, and importantly, the relative benefit vs. placebo remained across the different systolic blood pressure categories (<110 mmHg: HR 0.76; 95% CI 0.60–0.97, ≥110–<120 mmHg: HR 0.76; 95% CI 0.57–1.02, ≥120–<130 mmHg: HR 0.81; 95 CI 0.61–1.08; ≥130 mmHg: HR 0.67; 95% CI 0.51–0.87, P for interaction = 0.78) [56].

In the trial, 23.4% of patients were women. Dapagliflozin reduced the risk of the primary outcome to a similar extent according to gender (men: HR 0.73; 95% CI 0.63–0.85, women: HR 0.79; 95% CI 0.59–1.06, P for interaction = 0.67). Similarly, other efficacy outcomes, improvement in symptoms, and the safety profile of dapagliflozin did not differ according to gender [57].

Atrial and ventricular arrhythmias were particularly analyzed in the DAPA-HF trial, as these patients are at risk for such events [29]. At baseline, 40.3% of patients had a history of atrial fibrillation. The history of atrial fibrillation did not modify the benefits of dapagliflozin vs. placebo regarding the primary outcome (with atrial fibrillation: HR 0.75; 95% CI 0.62–0.92; without atrial fibrillation: HR 0.74; 95% CI 0.62–0.88; P for interaction = 0.88). The benefits of dapagliflozin vs. placebo regarding the components of the primary outcome, all-cause mortality, and improvement of symptoms remained independent of atrial fibrillation status [58]. Regarding ventricular arrhythmias, dapagliflozin reduced the incidence of the first occurrence of any serious ventricular arrhythmia, resuscitated cardiac arrest, or sudden death compared to placebo (HR 0.79; 95% CI 0.63–0.99) [59].

With respect to other comorbidities, an ‘obesity survival paradox’ was observed in the DAPA-HF trial, with fewer events in those patients with obesity class I compared to patients with normal weight, underweight, or obesity class ≥2. However, the effect of dapagliflozin vs. placebo on the primary outcome did not change according to baseline body mass index: under/normal weight: HR 0.74; 95% CI 0.58–0.94, overweight: HR 0.81; 95% CI 0.65–1.02, obesity class I: HR 0.68; 95% CI 0.50–0.92, obesity class II/III: HR 0.71; 95% CI 0.51–1.00; (P for interaction = 0.79). The same was observed for other outcomes. In addition, dapagliflozin reduced body weight by 0.9 kg at 8 months; *p* < 0.001 [60]. In the DAPA-HF trial, 12.3% of patients presented chronic obstructive pulmonary disease at baseline. Although the incidence rates of the primary outcome were higher in these patients, the benefit of dapagliflozin was not modified by the history of this comorbidity (with: HR 0.67; 95% CI 0.48–0.93, without: HR 0.76; 95% CI 0.65–0.87, P for interaction = 0.47) [61]. Iron deficiency, defined as a ferritin level < 100 ng/mL or a transferrin saturation < 20% and a ferritin level 100 to 299 ng/mL, was observed in 43.7% of participants in the DAPA-HF trial. These patients had a higher risk of events, but the effect of dapagliflozin on the primary outcome was consistent, regardless of the presence of iron deficiency (with: 0.74; 95% CI 0.58–0.92; without: HR 0.81; 95% CI 0.63–1.03, P for interaction = 0.59). Similar trends were observed regarding other outcomes [62].

Several studies have analyzed the impact of background HF therapy on the efficacy and safety of dapagliflozin in the DAPA-HF trial. Thus, a study that analyzed the use of furosemide or equivalent at baseline (no diuretic, <40 mg, 40 mg, and >40 mg daily) showed that the relative efficacy of dapagliflozin did not differ across the different subgroups (HR 0.57; 95% CI 0.36–0.92, HR 0.83; 95% CI, 0.63–1.10, HR 0.77; 95% CI 0.60–0.99, and HR 0.78; 95% CI, 0.63–0.97, respectively, P for interaction = 0.61) [63]. Another study analyzed whether the benefits of dapagliflozin were consistent in relation to background HF therapy (yes/no): mineralocorticoid receptor antagonist, sacubitril/valsartan, ivabradine, diuretic, digoxin, implanted cardioverter-defibrillating device, and cardiac resynchronization therapy and also according to the dose (≥50% and <50% of target dose): ACEi/ARB, beta blocker, mineralocorticoid receptor antagonist, showing that the benefit of dapagliflozin was independent of background HF therapy [64]. As sacubitril/valsartan is one of the pillars for the treatment of patients with HFrEF, a specific sub-study was developed. At baseline, 10.7% of patients were taking sacubitril/valsartan. Baseline treatment with sacubitril/valsartan did not modify the beneficial effects of dapagliflozin vs. placebo (with: HR 0.75; 95% CI 0.50–1.13; without: HR 0.74; 95% CI 0.65–0.86, P for interaction = 1.0). Similar findings were observed for the rest of the outcomes. Importantly, no more episodes of hypovolemia or hypotension were observed with the concomitant use of sacubitril/valsartan and dapagliflozin [65]. Although mineralocorticoid receptor antagonists represent another pillar of treatment for patients with HFrEF, their use is lower when compared to renin-angiotensin system inhibitors or beta blockers, likely due to safety concerns, mainly hyperkalemia [1,13,15]. In the DAPA-HF trial, 71% of patients were treated with a mineralocorticoid receptor antagonist. These patients were younger and had worse NYHA functional class and lower left ventricular ejection fraction, but better renal function. However, the relative efficacy of dapagliflozin did not differ according to its use (with: HR 0.74; 95% CI 0.63–0.87, without: HR 0.74; 95% CI 0.57–0.95, P for interaction = 0.97). The same was observed for other endpoints. No additional safety concerns were observed with the concomitant use of dapagliflozin [66]. Remarkably, those patients randomized to dapagliflozin had half the incidence of moderate/severe hyperkalemia associated with mineralocorticoid receptor antagonists use, compared to placebo (1.4% vs. 2.4%; HR 0.50; 95% CI 0.29–0.85). This offers a clear advantage, as more patients with HFrEF would be able to take mineralocorticoid receptor antagonists [67]. Finally, the benefits of dapagliflozin were independent of background glucose-lowering therapy (P for interaction 0.39 for the primary endpoint) or the type used, among those patients with type 2 diabetes from the DAPA-HF trial [68].

Regarding events, an additional prespecified exploratory outcome was the primary outcome plus worsening HF symptoms/signs leading to the initiation of new, or the augmentation of existing, oral treatment. In line with the primary endpoint of the study, this new combined endpoint was also significantly reduced by dapagliflozin [69]. Furthermore, dapagliflozin significantly improved symptoms, physical function, quality of life, and health status among patients with HFrEF [70]. Another important point was that in the DAPA-HF trial, the benefits of dapagliflozin occurred early after randomization, with a sustained statistically significant benefit over time. Importantly, patients with a recent HF hospitalization tended to benefit more from treatment with dapagliflozin [71]. These data clearly indicate that dapagliflozin should be prescribed early in those patients with HFrEF. Finally, an exploratory analysis estimated the projected long-term treatment effects of dapagliflozin in HFrEF patients, suggesting that the benefits of dapagliflozin remain over the duration of the patient’s lifetime [72].

### 3.2. Dapagliflozin and HFmrEF and HFpEF: The DELIVER Trial

The DELIVER trial was a phase 3, randomized, clinical trial that included 6263 patients ≥ 40 years with chronic HF and a left ventricular ejection fraction > 40%, with evidence of structural heart disease and elevated natriuretic peptides, with or without type 2 diabetes mellitus. Patients were randomized to receive dapagliflozin 10 mg daily or a placebo, in addition to the standard therapy. At baseline, the mean age was 72 years, 44% were women, 75% were in NYHA functional class II, and 45% had type 2 diabetes [43].

**Figure 4:**
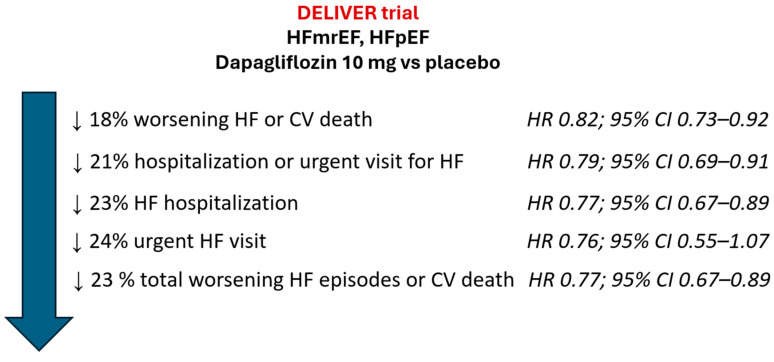
Main results of DELIVER trial. CI: confidence interval; CV: cardiovascular; HF: heart failure; HFmrEF: HF with mildly reduced ejection fraction; HFpEF: HF with preserved ejection fraction; HR: Hazard Ratio. Figure performed with data from reference [43].

After a median follow-up of 2.3 years, compared to placebo, dapagliflozin significantly reduced the risk of the primary combined endpoint of worsening HF (unplanned hospitalization for HF or an urgent visit for HF) or death from cardiovascular causes by 18% (HR 0.82; 95% CI 0.73–0.92). Similarly, dapagliflozin significantly reduced the risk of first or total worsening HF events (Figure 4). These results were independent of type 2 diabetes status. Importantly, the efficacy of dapagliflozin was similar across all the left ventricular ejection fraction spectrum. Remarkably, the incidence rates of renal adverse event (2.3%), volume depletion (1.3%), and major hypoglycemia (0.2%) was low with dapagliflozin and similar to the placebo group [43].

**Figure 5:**
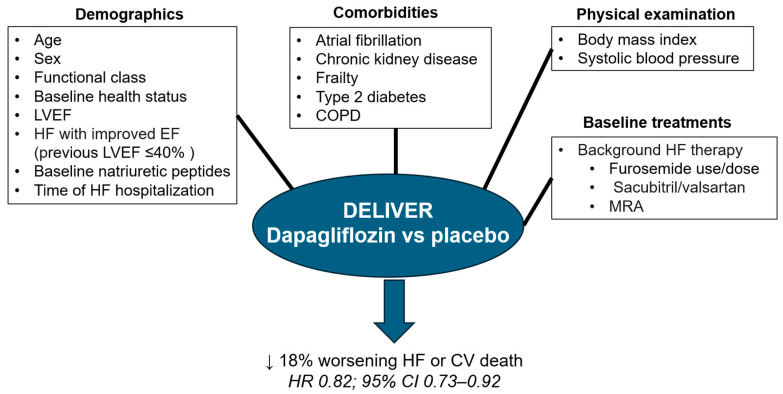
The relative benefits of dapagliflozin vs. placebo on the primary outcome in the DELIVER trial were independent of demographics, comorbidities, physical examination, and background therapies. CI: confidence interval; COPD: chronic obstructive pulmonary disease; CV: cardiovascular; HF: heart failure; LVEF: left ventricular ejection fraction; MRA: mineralocorticoid receptor antagonist; HR: Hazard Ratio. Figure performed with data from references [43,73,74,75,76,77,78,79,80,81,82,83,84,85,86,87,88,89,90].

Likewise, many prespecified sub-studies and post hoc analyses of the DELIVER trial have been published (Figure 5).

In the DELIVER trial, 1175 patients had normoglycemia, 1934 prediabetes (HbA1c 5.7 to < 6.5%), and 3150 type 2 diabetes (history of or HbA1c ≥ 6.5%) at baseline. The incidence rate of the primary outcome increased from 6.9 to 7.6 and 10.1 per 100 patient-years, respectively (*p* < 0.0001 for trend). Dapagliflozin reduced the risk of the primary outcome versus placebo in all subgroups (HR 0.77; 95% CI 0.57–1.04, HR 0.87; 95% CI 0.69–1.08, and HR 0.81; 95% CI 0.69–0.95, respectively, P for interaction = 0.82) [73].

In a prespecified analysis of the DELIVER trial, with 49% of patients with an eGFR < 60 mL/min/1.73 m^2^, the efficacy of dapagliflozin vs. placebo on the primary outcome was not modified by baseline renal function (eGFR ≥ 60 mL/min/1.73 m^2^: HR 0.84; 95% CI 0.70–1.00, eGFR 45–<60 mL/min/1.73 m^2^: HR 0.68; 95% CI 0.54–0.87 and eGFR < 45 mL/min/1.73 m^2^: HR 0.93; 95% CI 0.76–1.14, P for interaction = 0.16). In addition, dapagliflozin reduced the decline in renal function during the study (vs. placebo) [74].

The impact of age (mean age 71.7 years, 41.4% ≥ 75 years) on the efficacy and safety of dapagliflozin was also assessed in the DELIVER trial. The relative benefit of dapagliflozin on the primary outcome vs. placebo was independent of age (P for interaction = 0.95). Similar relative benefits were observed in other outcomes according to age. Adverse events were more common with age but without significant differences between groups [75]. In another study, the efficacy and safety of dapagliflozin according to frailty status were also analyzed. Although frail patients had a higher risk of events, frailty had no impact on the benefit of dapagliflozin over placebo regarding the primary endpoint (P for interaction = 0.40). Similarly, although side effects and treatment discontinuation were more frequent in frail patients, no significant differences were observed between groups [76].

In the DELIVER trial, low systolic blood pressure (<120 mmHg) was associated with higher HF and mortality events. Dapagliflozin modestly reduced systolic blood pressure by 1.8 mmHg within the first month of follow-up. The treatment effect of dapagliflozin on the primary endpoint was not affected by systolic blood pressure levels, nor adverse events [77]. In addition, in recently hospitalized patients with HF, initiation of dapagliflozin did not increase renal or hypovolemic serious adverse events and had minimal effects on blood pressure, suggesting that the early initiation of dapagliflozin was safe in this population [78].

Atrial fibrillation is a common condition in patients with HFpEF [29]. In the DELIVER trial, 18% of patients had paroxysmal atrial fibrillation, and 39% had persistent/permanent atrial fibrillation. Although the risk of events was higher among patients with atrial fibrillation, the benefit of dapagliflozin on the primary outcome was independent of the presence/type of atrial fibrillation (P for interaction = 0.49). Similar results were observed regarding other outcomes, such as HF hospitalization, cardiovascular death, all-cause mortality, and improvement of symptoms [79].

On the other hand, obesity was common in the DELIVER trial (normal weight 21.5%, overweight 33.1% and obesity 44.6%) and was associated with higher rates of HF hospitalization and worse health status. Whereas dapagliflozin reduced the risk of the primary outcome to a similar extent across all body mass index categories (P for interaction = 0.82), the improvement of symptoms was higher in patients with obesity. Treatment with dapagliflozin was associated with a modest reduction in body weight that was higher as body mass index increased [80].

Regarding background HF therapy at baseline, in the DELIVER trial, 10.9% of patients were not taking diuretics, 12.3% were on a non-loop diuretic, and 76.8% were taking loop diuretics at baseline. The efficacy of dapagliflozin on the primary endpoint was independent of the diuretic use (P for interaction = 0.64) or the loop diuretic dose (P for interaction = 0.57). Of note, treatment with dapagliflozin was associated with a significant reduction in the new initiation of loop diuretics (HR 0.68; 95% CI 0.55–0.84). In addition, loop diuretics dose decrease was more common in those patients assigned to dapagliflozin [81]. In the DELIVER trial, 42.6% of patients were taking mineralocorticoid receptor antagonists and 4.8% an ARNI at baseline. The relative efficacy of dapagliflozin over placebo about the primary endpoint was consistent, regardless of the use of mineralocorticoid receptor antagonists (P for interaction = 0.30) or ARNI (P for interaction = 0.75). No more adverse events were observed with the concomitant use of mineralocorticoid receptor antagonists or ARNI with dapagliflozin [82].

With regard to outcomes, in a prespecified analysis of the DELIVER trial that included those patients with HF with improved ejection fraction, defined as those whose ejection fraction improved from ≤40% to >40% (185 patients), these patients had the same benefit with dapagliflozin regarding the primary composite outcome, the first and total worsening HF events, and cardiovascular death, than patients with an ejection fraction consistently >40% [83]. The DELIVER trial also showed that dapagliflozin provided clinical benefit regardless of baseline NYHA functional class (P for interaction =0.921) and that also was associated with early and sustained improvements in NYHA functional class during the follow-up [84]. The benefits of dapagliflozin in the health status based on aggregate summary scores of the Kansas City Cardiomyopathy Questionnaire were also observed in the DELIVER trial. The greatest benefits were observed in domains related to symptom frequency and physical limitations [85]. A smaller clinical trial performed in patients with HFpEF has also shown improvements in patient-reported symptoms, physical limitations, and exercise function after only 12 weeks of treatment with dapagliflozin [86]. On the other hand, the benefits of dapagliflozin in the DELIVER trial were independent of baseline natriuretic peptide levels (P for interaction = 0.40 by quartiles and = 0.19 continuously for the primary outcome), but with a greater absolute benefit in those patients with higher NT-proBNP concentrations, suggesting the benefits of dapagliflozin even in more severe patients [87]. Furthermore, dapagliflozin did not only reduce the first HF event but the rate of total HF events (first and subsequent HF hospitalizations and urgent HF visits) and cardiovascular death, regardless of patient characteristics, in the whole spectrum of ejection fraction [88,89]. In addition, the benefits of dapagliflozin over placebo in the primary endpoint were irrespective of the time of HF hospitalization (recently hospitalized vs. patients without recent hospitalization; P for interaction = 0.71), with similar rates of adverse events between groups, suggesting that the early initiation of dapagliflozin, was effective and safe, even in patients with a recent HF hospitalization [90]. Similarly, to the DAPA-HF trial, in the DELIVER trial, the benefits of dapagliflozin occurred as early as within the first 2 weeks of treatment initiation and remained over time [91]. Finally, an exploratory analysis that estimated the projected long-term treatment effects suggested that treatment with dapagliflozin would extend event-free survival by up to 2.0 to 2.5 years among middle-aged and older individuals with HFpEF or HFmrEF [92].

### 3.3. Dapagliflozin in the Whole Spectrum of HF

**Figure 6:**
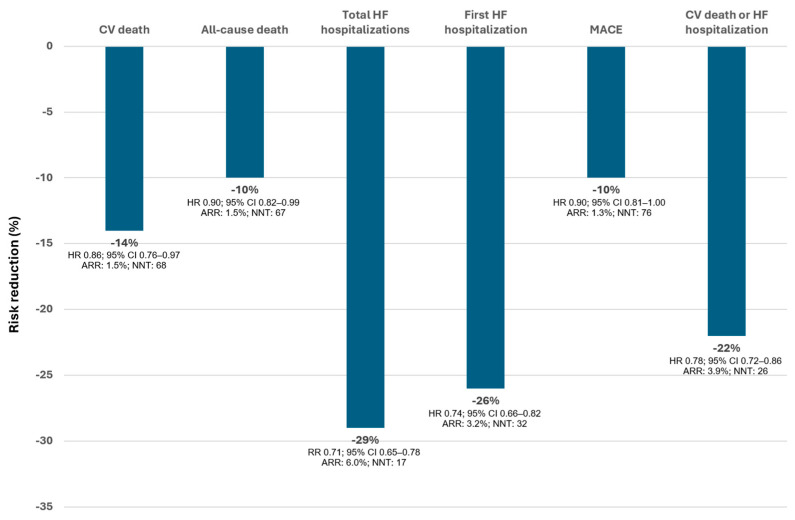
Risk reduction of events with dapagliflozin (vs placebo). Data from the pooled meta-analysis of the DAPA-HF and DELVER trials (median follow-up of 22 months). AAR: absolute risk reduction; CI: confidence interval; CV: cardiovascular; HF: heart failure; HR: Hazard Ratio; MACE: major adverse cardiovascular events (CV death, myocardial infarction, or stroke); NNT: number needed to treat over the median follow-up; RR: Rate Ratio. Figure performed with data from reference [93].

To answer this question, a patient-level, pooled meta-analysis using data from the DAPA-HF and DELIVER trials was performed, including a total of 11,007 patients (4744 had a left ventricular ejection fraction ≤ 40% and 6263 > 40%, 5503 received placebo and 5504 dapagliflozin). The median follow-up was 22 months. Overall, dapagliflozin reduced the risk of cardiovascular death by 14% (HR 0.86; 95% CI 0.76–0.97), death from any cause by 10% (HR 0.90; 95% CI 0.82–0.99), total HF hospitalizations by 29% (rate ratio 0.71; 95% CI 0.65–0.78) and MACE by 10% (HR 0.90; 95% CI 0.81–1.00) (Figure 6). Of note, dapagliflozin demonstrated a benefit not only in reducing HF outcomes but also in decreasing cardiovascular and total mortality. In addition, there was no evidence that the effect of dapagliflozin differed by ejection fraction (cardiovascular death: P for interaction = 0.94; all-cause death: P for interaction =0.58; total HF hospitalizations: P for interaction = 0.84; first hospitalization for HF: P for interaction =0.40; MACE: P for interaction = 0.93; cardiovascular death or HF hospitalization: P for interaction =0.71). In other words, the benefits of dapagliflozin can be extended to the whole spectrum of HF, regardless of the HF phenotype or ejection fraction [93]. This effect of dapagliflozin in the whole spectrum of HF was independent of gender [94]. Importantly, the benefits of dapagliflozin do not only extend to the whole spectrum of ejection fraction, but to the wide range of severity, as it has been observed that dapagliflozin also improves the maximal functional capacity even in patients with data of advanced HFrEF, and reduces circulating antigen carbohydrate 125 (CA125), a marker of fluid overload in HF [47,95].

The DAPA-HF and DELIVER trials showed in an outpatient setting that the clinical benefit of dapagliflozin started early after the initiation of the treatment; that this occurred among patients that had had a recent worsening HF episode, as well as in those patients with chronic HF. In addition, these studies also showed that the positive impact of dapagliflozin could persist over the duration of a patient’s lifetime [71,72,90,91,92]. However, recent data have also demonstrated that the positive impact of the treatment with dapagliflozin starts even during HF hospitalization, as early as it can be prescribed after the stabilization of the patient. Thus, a single-center, controlled, randomized study that included 102 patients over 18 years admitted to the hospital with acute HF, requiring intravenous administration of loop diuretics, showed that dapagliflozin was associated with a more marked weight loss and less need to increase diuretic therapy during hospitalization, without significant renal function deterioration [96]. Another multi-center, open-label, randomized study that examined the decongestive effect of dapagliflozin compared to the thiazide-like diuretic metolazone in patients hospitalized for HF and resistant to treatment with intravenous furosemide showed similar effectiveness at relieving congestion between dapagliflozin and metolazone, with more requirements of furosemide dose with dapagliflozin, but with less changes in plasma sodium and potassium and increases in urea and creatinine than with metolazone [97]. The DICTATE-AHF (Efficacy and Safety of Dapagliflozin in Acute Heart Failure; NCT04298229) trial examined the efficacy and safety of dapagliflozin on diuretic response, initiated within 24 h of hospital presentation, in 240 patients hospitalized for acute HF, requiring intravenous loop diuretics, with or without type 2 diabetes and an eGFR ≥ 25 mL/min/1.73 m^2^. Patients with type 1 diabetes, systolic blood pressure < 90 mmHg, serum glucose < 80 mg/dL, use of intravenous inotropic therapy, and history of diabetic ketoacidosis were excluded from the study [98]. Patients received dapagliflozin 10 mg once daily or structured usual care until day 5 or hospital discharge. Although not significant, there was a trend towards an improvement of the primary outcome (diuretic response, expressed as the cumulative change in weight per cumulative loop diuretic dose from enrolment to day 5 or discharge, if sooner) with dapagliflozin (OR 0.65; 95% CI 0.41–1.01, *p* = 0.06). However, dapagliflozin significantly increased both 24-h natriuresis (*p* = 0.025) and 24-h urine output (*p* = 0.005) and decreased both time to completing intravenous diuretic therapy (*p* = 0.006) and time to hospital discharge (*p* = 0.007). The incidence of adverse events (change in eGFR, inpatient mortality, symptomatic hypotension, total or serious hypoglycemia events, genitourinary infections, or severe hypokalaemia) did not differ between groups [99].

All these data clearly indicate that the early initiation of dapagliflozin, even during acute HF hospitalization is safe and may be associated with better outcomes.

## 4. Discussion

Although in this manuscript we have focused on the practical management of dapagliflozin among patients with HF, currently, two SGLT2i have been approved for use in patients with HF, regardless of ejection fraction, empagliflozin and dapagliflozin [42,43,100,101].

**Figure 7:**
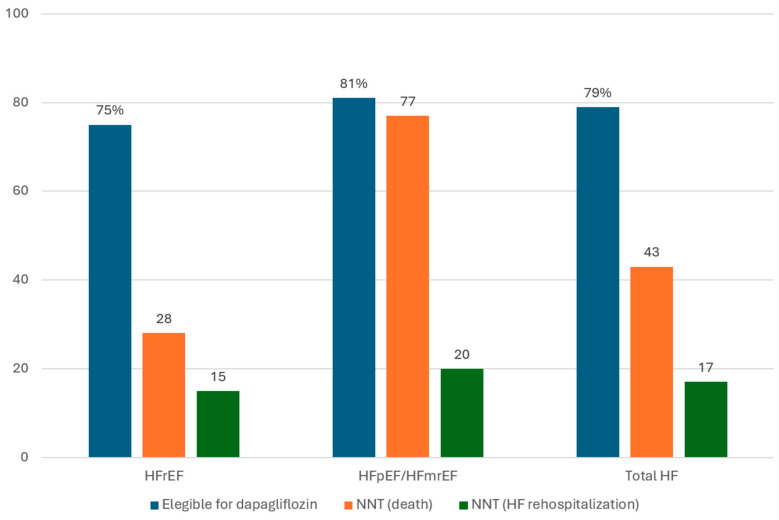
Projected effectiveness of dapagliflozin in patients with heart failure according to ejection fraction in clinical practice. HFmrEF: HF with mildly reduced ejection fraction; HFpEF: HF with preserved ejection fraction; HFrEF: HF with reduced ejection fraction; NNT: number needed to treat over one year of follow-up Figure performed with data from references [106,107,108].

Robust evidence from clinical trials and sub-studies has demonstrated that dapagliflozin reduces not only HF (re-)hospitalizations, but also cardiovascular mortality (NNT = 68) and all-cause death (NNT = 67) in the whole spectrum of HF, without a loss of efficacy according to ejection fraction [92]. On the other hand, different studies have shown that a great proportion of patients with HF in clinical practice are eligible for dapagliflozin according to the DAPA-HF and DELIVER criteria [102,103,104,105,106,107,108]. In addition, the full implementation of dapagliflozin in real-life populations would prevent/postpone new worsening HF events or even death [102,106,107,108]. For example, a Spanish registry of 5644 patients ≥50 years old consecutively hospitalized for HF in internal medicine departments in Spain showed that full implementation of dapagliflozin would lead to robust absolute risk reductions of mortality and HF readmission, with low NNT in patients with HF, regardless ejection fraction (Figure 7) [106,107,108].

These clinical beneficial effects of dapagliflozin rely on several pathophysiological mechanisms that have been investigated in different experimental and clinical studies. Thus, experimental studies have shown in different animal models with HF that dapagliflozin inhibits ventricular remodeling, attenuates cardiometabolic dysregulation and diabetes-induced diastolic dysfunction, reduces cardiac fibrosis and inflammation, improves aorta sympathetic tone and endothelial dysfunction [109,110,111,112,113,114,115,116,117,118]. The impact of dapagliflozin on cardiac remodeling has been confirmed in patients with chronic HF in the DAPA-MODA study, with significant improvements in left atrial volume, left ventricular geometry parameters and natriuretic peptides levels, after only 6 months of treatment [119].

From a practical point of view, since SGLT2i has little impact on different clinical variables (i.e., a modest effect on blood pressure and a slightly initial effect on renal function), in contrast to other HF drugs (i.e., beta-blockers: heart rate, blood pressure; renin-angiotensin system aldosterone inhibitors: renal function, potassium levels, blood pressure), this simplifies the use of dapagliflozin in clinical practice as it can also be easily prescribed in more complex patients, such as those with low heart rate, low blood pressure, or renal dysfunction [120,121]. Moreover, the addition of dapagliflozin may facilitate the introduction of other HF drugs, as it may reduce the risk of adverse events (i.e., hyperkalemia with mineralocorticoid receptor antagonists is reduced with the concomitant use of dapagliflozin) [67].

On the other hand, several studies have demonstrated the cost-effectiveness of dapagliflozin in the whole spectrum of HF patients, regardless of the healthcare system of different countries [122,123,124,125,126,127,128]. In fact, the immediate initiation of dapagliflozin provides greater clinical benefits [128]. A recent study has shown that in patients with HFrEF, dapagliflozin may be more cost-effective than empagliflozin mainly due to the specific beneficial effect of dapagliflozin on mortality [129]. In fact, although guidelines recommend the use of SGLT2i in the management of HF, independently of ejection fraction, it seems that there could be some difference between SGTL2i [130].

Despite the robustness of evidences with dapagliflozin, there are ongoing clinical studies that will provide further information that will extend the use of dapagliflozin in different clinical scenarios, such as the DAPA ACT HF-TIMI 68 trial, an investigator-initiated, clinical trial in patients with HF who have been stabilized during hospitalization for acute HF, that is evaluating the effect of in-hospital initiation of dapagliflozin on the clinical outcomes of cardiovascular death or worsening HF (NCT04363697) or the DAPA-MI that is assessing the effects of dapagliflozin on cardiometabolic outcomes in patients with myocardial infarction and evidence of impaired regional or global left ventricular systolic function or definite evidence of Q wave on electrocardiogram (NCT04564742).

## 5. Conclusions

HF is associated with great morbidity and mortality. The early initiation of those drugs that have been demonstrated to modify the clinical course of HF is mandatory to reduce HF burden. In light of recent evidence, clinical practice guidelines strongly recommend (recommendation IA) the use of SGLT2i in all patients with HF, regardless of the HF phenotype. Dapagliflozin has been demonstrated to reduce not only HF hospitalizations or HF worsening episodes but also mortality, with long-term sustained benefits in the whole spectrum of HF.

## Data Availability

Not applicable.
